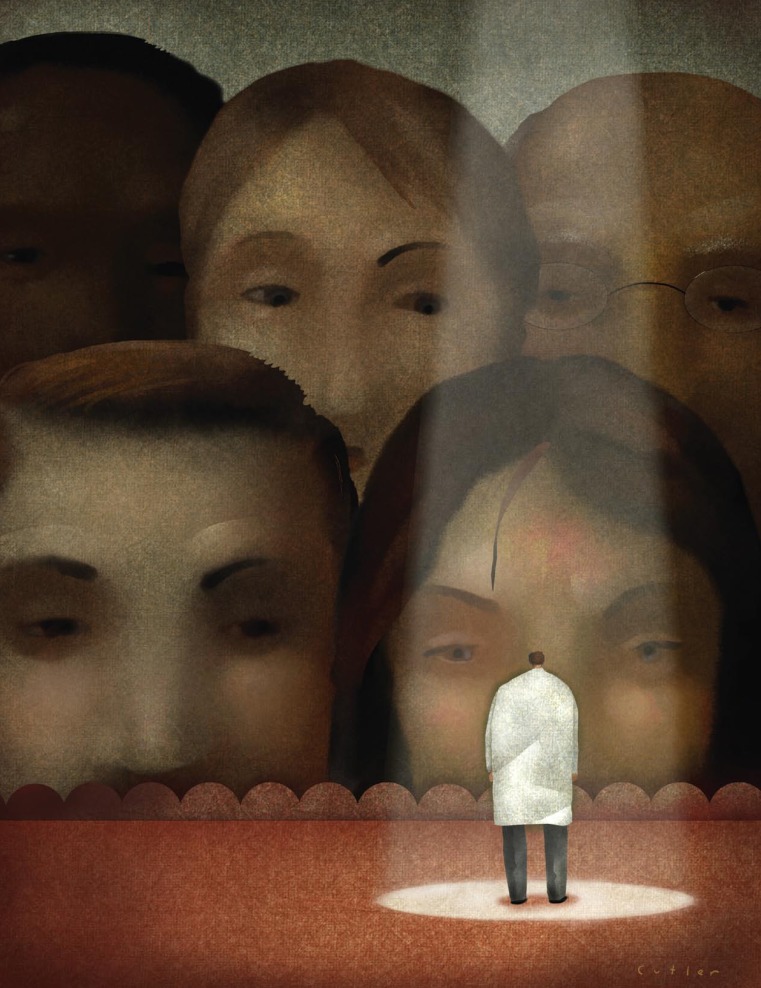# Outside Looking In: Understanding the Role of Science in Regulation

**DOI:** 10.1289/ehp.117-a104

**Published:** 2009-03

**Authors:** Tim Lougheed

When Rachel Carson took on the task of defining environmental health advocacy in the early 1960s, she made the business of government oversight look simple and straightforward. “Much of the necessary knowledge is now available, but we do not use it,” she wrote in her 1962 book *Silent Spring*. “We train ecologists in our universities and even employ them in our government agencies, but we seldom take their advice. We allow the chemical death rain to fall as though there were no alternative, whereas in fact there are many, and our ingenuity could soon discover more if given opportunity.”

Carson would likely be dazzled by the extent to which governments of all stripes have since called on scientific experts to help populate a regulatory galaxy that extends from the humblest of municipal bailiwicks to the global economy. Nevertheless, the unfolding of this new world of prevention and protection has been neither tidy nor consistent. The scientific community generates volumes of data about potential hazards to human health, but the process of interpretation—resulting ultimately in the development of policy—is often heavily shaped by political, economic, and even cultural interests, which can vary dramatically from one hazard to the next, as well as from one jurisdiction to the next.

The outcome of any regulatory deliberation can therefore be unexpected and downright frustrating. A given agent might be labeled a toxic threat in one place while being tolerated without prejudice somewhere else, even as the architects of each policy looked at the very same data. That prospect might puzzle many thoughtful and earnest observers who believe definitive scientific findings should yield equally definitive responses.

Daniel Sarewitz recalls his early days as a Congressional Science Fellow in 1989. “The scales fell from my eyes after about a week of being [in Washington, DC],” he says. “When you’re a scientist working in academia, what you see is scientists arguing about difficult problems to try to arrive at the truth. When you’re on the Hill, you realize what’s really going on is these problems are complicated both in terms of the science and in terms of the values. It’s possible to bring many different scientific lenses—interpretations of data, choices of what data to use, what theories to use—to any given complex problem. Not surprisingly, those choices end up mapping onto value preferences and political preferences.”

Today Sarewitz is director of the Consortium for Science, Policy, & Outcomes, which, in its own words, seeks to enhance the capacity of public policy to link scientific research to beneficial societal outcomes. But Sarewitz says the consortium faces resistance in moving these perspectives into open public debate. The difficulty, as he outlined in an article in the October 2004 issue of *Environmental Science & Policy*, stems from a common desire of both advocates and opponents of any given regulation to invoke science to make their respective cases. The former will insist that current knowledge warrants doing something, while the latter point to uncertainties in that same knowledge as justification for doing less, or perhaps nothing.

## A Case in Point

The example of bisphenol A (BPA) has recently testified to the ever more intricate subtleties of the regulatory review process. This organic compound is a building block in a number of widely used polymers, including the protective coating applied to the inside of food cans and the plastics used to form beverage containers such as baby bottles. Agencies in North America and Europe have regularly considered the potential health implications of this product ever since it entered commercial use more than 50 years ago.

Although various animal models raised questions about specific hazards—such as altering hormonal balances in rats—regulators generally concluded that any effect on humans was too small to yield measurable effects. Canada broke with international consensus in 2008, however, when it declared BPA toxic under its 1999 Canadian Environment Protection Act (CEPA), which applies to activities of two key federal departments, Environment Canada and Health Canada. “We have concluded that early development is sensitive to the effects of bisphenol A,” stated Health Minister Tony Clement in his formal announcement of the government’s move. “Although our science tells us exposure levels to newborns and infants are below the levels that cause effects, it is better to be safe than sorry. And so, if no new, relevant, and compelling information comes forward during the public consultation period, it is our intention to ban the importation, sale, and advertising of polycarbonate baby bottles.”

Meanwhile, in 2006, the European Food Safety Authority (EFSA), an independent risk assessment agency under the European Union, had conducted its own evaluation of BPA and concluded that, for the general population, exposure to the chemical was well below the tolerable daily intake (TDI), or the highest dose that can be tolerated every day without adverse effects. This TDI was based on the findings of several toxicity studies in rats, which together had yielded a specific NOAEL (no-observed-adverse-effect level, or the highest dose at which an adverse effect is not seen).

In July 2008, EFSA re-examined the safety of BPA, focusing on the possible differences between neonates and adults (in both humans and rats) in eliminating BPA from the body. EFSA’s scientific panel concluded that neonates are sufficiently capable of eliminating BPA from the body and that, because of metabolic differences, rats are more exposed to BPA than are humans. Shortly before Canada declared BPA toxic, EFSA made a public statement contending that the existing body of data on BPA did not warrant such action on its part.

The U.S. Food and Drug Administration (FDA), for its part, made specific reference to that EFSA observation and to a similar report from the Japanese National Institute of Advanced Industrial Science and Technology in reaching its own conclusions about the safety of BPA. “Each of these documents considered the question of a possible low-dose effect and concluded that no current [human] health risk exists for BPA at the current exposure level,” stated an agency release issued in spring 2008. The FDA declared BPA safe in a draft assessment released 15 August 2008, but by October, a subcommittee of the FDA Science Board issued a report challenging the agency’s conclusions, describing the assessment’s defined margin of safety as inadequate.

Steven Hentges, whose perspective as a representative of the American Chemistry Council became part of the official record of an October 31 meeting of the board, rejects this characterization. “This definitive conclusion and other similar statements in the report do not appear to be based on a sound and thorough scientific analysis, and in particular, one that follows the subcommittee’s own recommendations,” he said.

## What’s the Bottom Line?

The debate around BPA could look arcane to a parent who simply wants to know if baby’s bottle is at all harmful. For George Enei, acting director general of Environment Canada’s Science and Risk Assessment Directorate, the issue sits within a larger bureaucratic context. “Most people ask the question ‘How can a chemical substance be toxic in Canada but not in the United States or vice versa?’” he says. “The basic answer is that, while each country is following the same scientific methods and protocols, our respective legislation is different.”

Enei explains that whereas the United States combines various processes of environmental health assessment and management, Canada has separated them into three steps under CEPA. “We conduct a risk assessment that assembles all of the available science to determine if a substance is harmful or has the potential to cause harm to humans or the environment,” he says. “If the answer is yes, the substance is added to the list of toxic substances. Once the substance is on the list, we have access to a variety of options under CEPA to manage the risks that the assessment has identified. These include pollution prevention plans, regulations, or, in extreme cases, eliminating the substance from being used or brought into Canada.”

Enei credits Canada with taking these efforts further with its Chemicals Management Plan, leading the international community in a comprehensive sorting of no fewer than 23,000 chemicals that could pose some kind of threat to the environment or human health. Since the launch of this initiative in 2006, the United States and European Union have followed suit with similar programs: the U.S. Environmental Protection Agency’s (EPA) Chemicals Assessment and Management Program (ChAMP) and the European Commission’s Registration, Evaluation, Authorisation and Restriction of Chemical Substances (REACH) system. The three programs are equally ambitious, addressing tens of thousands of agents old and new.

BPA had been among 200 chemicals singled out by the Chemicals Management Plan for inclusion in a “challenge to industry,” meaning these chemicals were flagged as the most likely to cause environmental and/or health problems, and their manufacturers would be among the first to be asked about how these substances were being handled. Also, under provisions of CEPA, screening assessments examine scientific information and develop conclusions by incorporating weight-of-evidence and precautionary approaches.

Barbara McElgunn, health policy officer with the Learning Disabilities Association of Canada, has been examining the scientific findings around BPA and developmental neurotoxicity for years. “This is the first time in my memory that Canada has really taken the lead on any chemical in terms of regulation,” she says, noting that all too often regulatory policies have lagged behind decisions already made in the United States.

That might also have happened with respect to BPA, she adds, but the Chemicals Management Plan appears to have added momentum to Canada’s work in this field. By setting a fresh agenda that transcends the mandates of existing government departments, this new program managed to bring at least one longstanding debate to the fore.

“The tipping point is public political concern,” McElgunn says. “When you raise public political concern, then it seems that regulators can take action. If there’s no public concern and only scientific concern, it’s a different kettle of fish, unfortunately.”

## Complexity and Competing Interests

In the view of Peter Andrée, a political scientist at Carleton University in Ottawa, government actors will incorporate other factors outside of scientific evidence into their decisions, regardless of how much respect they have for the pertinent science. In this way he explains discrepancies in bureaucratic attitudes toward recombinant bovine somatotropin (rbST, also known as recombinant bovine growth hormone, rbGH) a synthetic version of a protein produced in the pituitary glands of cattle that enhances milk output.

The FDA sanctioned the use of rbST across the United States in 1993, while Health Canada eventually banned it in 1999, despite the findings of an independent review panel that there was “no biologically plausible reason for concern about human safety if rbST were to be approved for sale in Canada.” That panel’s only proviso was an rbST oral toxicity study in rats that resulted in a single test animal developing an antibody response at low dosage.

In its 1999 *Report on the Food and Drug Administration’s Review of the Safety of Recombinant Bovine Somatotropin*, the FDA wrote that “such response was consistent with that produced by a number of food proteins and is not necessarily an indication of absorption of intact rbGH. As rbGH produces significant biological effects when injected into rats, this study supported the inability of rbGH to cause significant biological effects following oral administration even at doses 50 times greater than the injected dose.” The FDA then suggested that “the Canadian reviewers did not interpret the study results correctly and that there are no new scientific concerns regarding the safety of milk from cows treated with rbGH.”

For his part, Andrée regards this exchange from a different perspective. “My tendency is to look first towards the political economy of the issue,” he says. More specifically, he notes significant distinctions between each country’s dairy industry. The larger, corporation-centered U.S. producers would welcome the prospect of getting more milk from the same number of cows, he says. Sentiments tended the other way in Canada, where more of those producers are family-run operations.

“There’s a whole supply management system that keeps farm sizes reasonably small and allows them to be profitable,” explains Andrée, adding that these producers would be more likely to lobby the Canadian government to restrict rbST, thereby maintaining the existing level of milk output within a protected market. Any ban would thus win favor from an organized political constituency while allowing government administrators to claim they are championing public health, regardless of scientific testimony that little or no hazard existed.

The urgency to act grows once a hazard has actually been demonstrated, even if the nature of the hazard is not yet fully understood, says Manolis Kogevinas, an epidemiologist with the Center for Research in Environmental Epidemiology in Barcelona. That has been the case in Europe, where the impact of bovine spongiform encephalopathy (BSE) continues to reverberate. As thousands of animals were diagnosed with this condition, politicians initially found themselves defending the safety of meat, offering assurances that the best science of the day had found no implications for human health. Then, when further inquiry revealed that BSE was caused by a strange class of mis-folded proteins called prions, many of these same politicians had to do a very public about-face, acknowledging that a link with human health might exist.

The BSE experience has unquestionably shaped the European outlook on environmental health regulation, says Kogevinas. In particular, EU lawmakers have adopted a critical stance toward claims associated with the use of genetically modified organisms (GMOs) in food and pharmaceutical processing. In 1998, this ongoing suspicion threatened to undermine an economic pillar of Switzerland, where voters were offered the opportunity to all but outlaw genetic research on plants and animals within the country. The defeat of this referendum was a boon for Swiss-based drug giants such as Novartis and Hoffmann-La Roche, which depend heavily on their ability to engage in this kind of research. Had this measure passed, such work and the people conducting it likely would have migrated elsewhere, hollowing out a mainstay of the Swiss economy.

Nor did this 1998 decision put the matter to rest. In 2005 another referendum successfully installed a five-year moratorium on the use of GMO products in Swiss agriculture. Although this latest decision does not have a direct impact on laboratory work, organizations such as the Swiss Biotech Association and Swiss Trade Association have voiced their fears that such legislative maneuvers will restrict the freedom of researchers, fostering an international perception of their nation as one that is unfriendly to scientific activity.

Kogevinas concedes that the hue and cry over GMOs has no counterpart in North America. He suggests that this distinction reflects a more fundamental difference in the nature of public engagement, particularly the manner in which administrative procedures are executed. “In North America you have a much more structured and transparent system for contact with institutions, with organizations, with communities,” he says. “We have less of a tradition of that in Europe.” In fact, new rules have opened up EU regulatory review committee meetings that formerly provided little or no public access.

Still, says Kogevinas, having long been excluded from such proceedings, many members of the scientific community have little appetite for contributing to the development of public policy except in the most technical manner. That reluctance can play into the strategies adopted by many policy makers, as Kogevinas discovered when he waded into Spanish deliberations over the approval of municipal garbage incinerators, a potential source of dioxin emissions.

“The government asks certain questions as if they were scientific questions, when actually they are political questions, and sometimes we mix things up and try to respond to political questions using strictly scientific criteria,” he explains. The real question, he says, is not whether dioxin emissions should be limited to 0.1 ng/m^3^ or some other value. Instead, he says, “the real question is ‘What do we do with all the residues?’ The real question is whether we need a particular incinerator—or incinerators in general—as a means of waste reduction.”

## Precautionary Tales

Similar distractions crop up in U.S. discussions, whether the participants are looking at incinerators, stem cells, or GMOs. What often sets European conclusions apart, however, is commitment to a tenet known as the precautionary principle.

The precautionary principle—another way of saying “better safe than sorry”—has been making its way into the regulatory practices of governments since the 1930s. By the 1990s, major events such as the Rio de Janeiro Earth Summit began casting the idea in legal language. In 1998, the Science and Environmental Health Network convened an international gathering of scientists, philosophers, lawyers, and environmental activists at the Johnson Foundation Wingspread Conference Center in Wisconsin, yielding this succinct definition of the principle: “When an activity raises threats of harm to the environment or human health, precautionary measures should be taken even if some cause-and-effect relationships are not fully established scientifically.”

Members of the European Union took note, and within two years had adopted a similar statement as the foundation for EU environmental regulation. The result has imposed the juridical equivalent of “guilty until proven innocent” on any manufacturer seeking permission to introduce a product into the marketplace, demanding that public protection from potential harm be placed ahead of commercial interests.

Such a requirement accounts for much of the administrative distance that separates Europe from the United States, according to John Bucher, associate director of the National Toxicology Program (NTP), a multiagency toxicology and testing program housed at the National Institute of Environmental Health Sciences. “The U.S. system has evolved around the supposition that the government is responsible for providing information, or utilizing and acting on information supplied by industry, that would suggest that a particular chemical shouldn’t be used in commerce,” he says. In other words, precaution is not imposed upon commercial interests as a default position; instead, hazards and risks are defined by government on a case-by-case basis.

Bucher suggests that although regulatory agencies evaluate the risks of agents fairly uniformly, they may pay more attention to cases that rise to political or social prominence. Nor can he and his colleagues in the NTP sway this tendency, since they lack the mandate of a regulatory agency and cannot define or assess risk as part of their observations. “So we must couch things in terms of whether we have concerns over a particular level of human exposure that’s going on in the population,” he says. “We do however, select agents for evaluation through our programs . . . that we feel warrant regulatory agency consideration.”

The precautionary approach in the United States has been further stalled by a modest rider added to a congressional spending bill in 2001—two sentences with no official name but known alternately as the Information Quality Act or the Data Quality Act. This seemingly minor legislation has become contentious for requiring federal agencies to optimize the “quality, objectivity, utility, and integrity” of the information supporting regulatory activities.

Critics have highlighted this stipulation as a loophole for corporations to hold up the implementation of restrictions on their products. For Chris Mooney, author of the 2005 book *The Republican War on Science*, this move represented “an unprecedented and cumbersome process by which government agencies must field complaints over the data, studies, and reports they release to the public. It is a science abuser’s dream come true.”

On the other hand, when the EPA considered the endocrine-disrupting potential of the herbicide atrazine in 2003, the Data Quality Act was invoked to clarify the experimental methods that were being used to argue that such effects from this agent had been observed in frogs. “Publication of a research article in a peer-reviewed scientific journal does not mean that the research has been accepted as valid by the scientific community and that it should be considered reliable for regulatory purposes,” argued members of the Center for Regulatory Effectiveness (CRE) in correspondence published in the January 2004 issue of *EHP*. A self-styled regulatory watchdog, the CRE applauded the introduction of the legislation as a brake against an overzealous embrace of preliminary or incomplete research findings.

That said, the precautionary principle is not altogether absent from U.S. regulations. In 2000, the FDA submitted a paper on its national food safety system to the Organisation for Economic Co-operation and Development, complete with an annex illustrating the role that precaution plays in the system. This document outlined various adjustment factors that would be applied to account for prospects such as sensitive subgroups within a tested population, extrapolation from short-term study data to assess chronic effects, extrapolation from animal data to human application, and variations within a human population, such as age or sex.

Still, the enforcement of precaution can raise new challenges that scientists do not yet know how to meet. Early in 2009, the European Parliament was putting the finishing touches on regulations banning chemicals that go into some of the world’s most widely used pesticides. Richard Tren, director of the nonprofit organization Africa Fighting Malaria, insists that the ban will prompt many nations on that continent to abandon their use of pesticides that are effectively and safely managing a public health scourge.

“This is a victory for the environmental lobby and a defeat of sound science,” he wrote in the 20 January 2009 edition of the Vancouver newspaper *The Province*. “Sensible regulations should evaluate the risks posed by chemicals to humans and the environment based on sound scientific evidence. Regulators shouldn’t be solely concerned with whether a pesticide is hazardous in the lab. Most important is how the pesticide is used and how diluted the active ingredient is.”

Observers such as McElgunn take another view. “Dilution of toxicants is not a solution, even when all the science is in and we have a NOAEL for sensitive end points,” she says. “Others would argue that sound scientific evidence should include both low-dose testing to uncover endocrine effects and neurodevelopmental effects, and cumulative assessments of substances that have similar properties or that produce similar adverse effects.”

For her part, Rachel Carson did not dismiss the use of chemical products, only their indiscriminate use—and she charged policy makers with the responsibility to discriminate. But weighing the scientific evidence in fulfillment of that responsibility is no simple task. According to Sarewitz, policy makers must deal with the harsh reality that there is not always one best way to use chemical compounds, which is why it can be so difficult to identify and eliminate toxicants. “We’re very glib about how easy that task is,” he says, “both in terms of the question of identifying what things do and the costs and consequences of getting rid of stuff.”

## Figures and Tables

**Figure:**